# Motility of Different Gastric *Helicobacter* spp.

**DOI:** 10.3390/microorganisms11030634

**Published:** 2023-03-01

**Authors:** Rama Bansil, Maira A. Constantino, Clover Su-Arcaro, Wentian Liao, Zeli Shen, James G. Fox

**Affiliations:** 1 Department of Physics, Boston University, Boston, MA 02215, USA; 2 Department of Biological Engineering, Massachusetts Institute of Technology, Cambridge, MA 02138, USA

**Keywords:** flagella, *Helicobacter*, motility, unipolar bacteria, bipolar bacteria, gastric mucin, *H. pylori*, *H. suis*, *H. cetorum*

## Abstract

*Helicobacter* spp., including the well-known human gastric pathogen *H. pylori*, can cause gastric diseases in humans and other mammals. They are Gram-negative bacteria that colonize the gastric epithelium and use their multiple flagella to move across the protective gastric mucus layer. The flagella of different *Helicobacter* spp. vary in their location and number. This review focuses on the swimming characteristics of different species with different flagellar architectures and cell shapes. All *Helicobacter* spp. use a run-reverse-reorient mechanism to swim in aqueous solutions, as well as in gastric mucin. Comparisons of different strains and mutants of *H. pylori* varying in cell shape and the number of flagella show that their swimming speed increases with an increasing number of flagella and is somewhat enhanced with a helical cell body shape. The swimming mechanism of *H. suis*, which has bipolar flagella, is more complex than that of unipolar *H. pylori. H. suis* exhibits multiple modes of flagellar orientation while swimming. The pH-dependent viscosity and gelation of gastric mucin significantly impact the motility of *Helicobacter* spp. In the absence of urea, these bacteria do not swim in mucin gel at pH < 4, even though their flagellar bundle rotates.

## 1. Introduction

**Figure 1:**
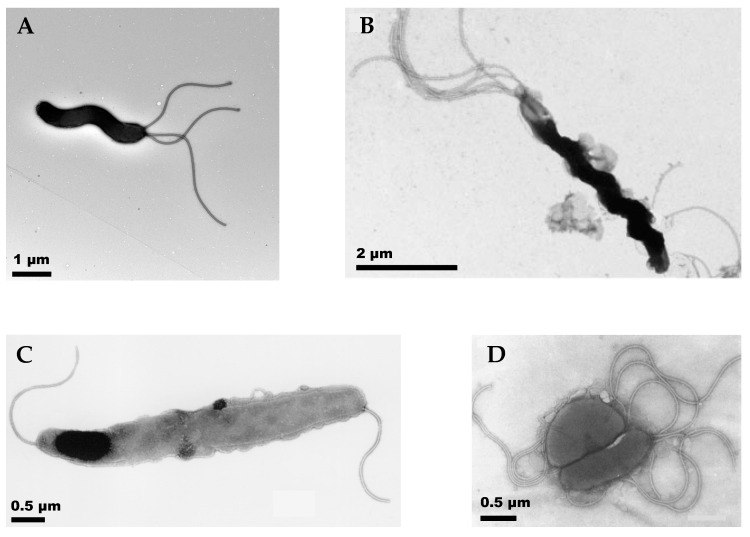
TEM images of different *Helicobacter* spp. showing flagella at different locations on the cell body. (**A**) *H. pylori*, scale bar 1 μm [4]. (**B**) *H. suis*, scale bar 2 μm. The flagella at the lower end are not fully visible in the image [5]. (**C**) *H. cetorum*, scale bar 0.5 μm [6]. (**D**) *H. mustalae*, scale bar 0.5 μm [7]. The images are reproduced from references [4,5,6,7] under Creative Commons Attribution 4.0 International Licenses.

**Helicobacters.** Helicobacters are a common Gram-negative helical-shaped gastric pathogen known to naturally infect not only humans but many other mammals, causing gastric diseases in their hosts. In addition, the human gastric pathogen *H. pylori* is associated with gastric cancer [1]. About 35 *Helicobacter* spp. have been identified, including both enterohepatic and gastric species. Some of the non-pylori *Helicobacter* spp. have been shown to transfer to humans, causing or exacerbating gastric diseases, and have also been implicated in other non-gastric illnesses [2,3]. The non-pylori *Helicobacter* spp. differ from *H. pylori*, exhibiting differences in cell shape and helicity, as well as in the location and number of flagella, and thus offer the potential to examine the influence of these factors on motility in the same bacterial genus. All gastric *Helicobacter* spp. have lophotrichous (i.e., multiple) sheathed flagella that can be confined to one or both poles (unipolar or bipolar) or be located on the side (lateral), as illustrated in Figure 1 which shows TEM images of a few examples. The TEM image of the *H. pylori* LSH 100 strain from humans [4] shows multiple unipolar flagella, *H. suis* from pigs [5] has multiple bipolar flagella, *H. cetorum* from whales and dolphins is also bipolar with a single flagellum at each pole [6], and *H. mustelae* from ferrets have both lateral and polar flagella [7]. The flagella vary in length and helicity among different species. The cell body shapes and sizes are also different, with *H. suis* having almost twice as many helical turns as *H. pylori*, and *H. cetorum* having a slightly helical, fusiform cell body that is tapered at both ends. Furthermore, the number of flagella and the helical cell body shape and size parameters also vary among different bacterial strains in the same species, as well as in individual bacteria from the same culture. 

In this review, we first address some of the general features relevant to the motility of flagellated bacteria such as *Helicobacter* spp. and then focus on the motility of three species, *H. pylori*, *H. suis*, and *H. cetorum*, for which detailed measurements and analysis of swimming mechanisms are available.

**Helicobacter flagellar and chemotaxis machinery.** Helicobacters have developed optimal motility and chemotaxis strategies to move across the highly viscoelastic and acidic environment presented by the mucus barrier to colonize the gastric epithelium (for a review see [8]). One of the key factors in determining their motility is the structure of the flagellum, a highly complex nanomachine made up of about 30 different proteins [9]. The flagellum of *H. pylori* is composed of multiple protein subunits, similar to many Gram-negative bacteria. It consists of three components: the basal body, hook, and filament. The C-ring, MS-ring, motor, and type III secretion systems form the complex basal body of the flagellum. To generate the high torque required for moving across highly viscoelastic media, the flagellar motor of *H. pylori*, like other ε-proteobacters such as *Campylobacter jejuni*, has adapted by having a large scaffold of proteinaceous, periplasmic rings to increase the radius of the contact lever point and to accommodate the large number of stators (17 for *C. jejuni* and 18 for *H. pylori*) that are present at high occupancy [10,11,12]. 

As in other flagellated bacteria, various flagellar proteins are involved in controlling the motility of *H. pylori*. FlgE is the main protein of the flagellar hook, and strains lacking the *flgE* gene show no motility. FlaA, FlaB, and FliD are the major components of the flagellar filament and are important for motility as well. Strains lacking the *flaA* and *flaB* genes exhibit truncated flagella with reduced motility in mouse models [13]. The *fla*A and *fla*B double-mutant strain was completely non-motile and lacked any form of flagella [14]. Mutation of the *fli*D gene results in non-motile bacteria with short flagella, which are unable to colonize the gastric mucosa of mice [15]. Mutations in related genes, *fliM*, *fliY*, *fliG*, and *motB*, cannot produce flagella or in some mutants, reduce flagellar motility. The direction of flagellar rotation is controlled by the chemotaxis-signaling proteins CheY and ChePep, and the flagellar rotor protein FliN; strains with mutations in the genes encoding these proteins exhibit reduced flagellar motility [16,17,18]. 

The chemotaxis of *H. pylori* involves a cascade of protein interactions, as described in the review by Lertsethtakarn et al. [19]. Briefly, four chemoreceptors called *Transducer Like Proteins*, TlpA, TlpB, TlpC, and TlpD, sense the environment and move the bacterium towards a neutral pH environment. The receptor TlpA is activated by the chemoattractants arginine and bicarbonate [20], and TlpB and TlpD are the pH-sensing receptors that move the bacterium away from an acidic environment [21,22]. Urea serves as a chemoattractant whose degradation by urease is crucial for the growth and motility of *H. pylori* in the acidic environment of the gastric mucosa [23]. The chemoreceptor TlpB is also involved in sensing urea [24]. Various Che proteins are involved in transmitting the signal from the Tlp chemoreceptors to the flagella, enabling the bacterium to move in response to the chemical gradient [19]. 

Unlike *H. pylori*, the flagellar gene structure and functions of *H. suis* and *H. cetorum* are not well studied. However, all flagella-subunits and assembly genes are conserved in the three gastric *Helicobacter* spp. discussed in detail in this review. Gastric *Helicobacter* genomes encode flagellar sheath adhesin (HpaA), a protein that protects flagellin subunits from depolymerization in low pH environments. When comparing the proteins encoded in the genomes of Helicobacters that have flagellar sheaths (FS+) against Helicobacters that have unsheathed flagella (FS-), the homologs of nearly 40 proteins were found significantly more frequently in the FS+ Helicobacter species compared to FS- Helicobacter species. Four of these proteins, HP1486, HP1487, HP1488, and HP1489, were found exclusively in the FS+ Helicobacter species and appear to be part of a tripartite efflux system [25]. A comparative genomics analysis to identify potential metabolic and virulence gene potentials in gastric versus enterohepatic Helicobacter species has been discussed [26]. 

**Swimming mechanisms of flagellated bacteria.** The swimming of polarly flagellated bacteria is more complex than the run-tumble, random walk mechanism originally postulated by Berg based on the swimming of *E. coli* bacteria, which have peritrichous flagella distributed over the entire body [27]. In run-tumble swimming, the flagella rotate in a counterclockwise direction, forming a bundle, and the bacterium swims in a given direction (commonly referred to as a “run”). When one or more flagella begin to rotate in the opposite direction they fall out of the bundle and the lack of coordinated flagellar motors causes the bacterium to tumble without much propulsion until the bundle reforms and the bacterium swims off in another, random direction. During a tumble, the cell undergoes an active reorientation that randomly changes the orientation of the next run. Runs in the favorable direction are extended to bias the random walk toward more-favorable environments. Alternative swimming strategies such as the run-reverse [28,29,30] and a three-step run-reverse-flick [31] have been observed in several bacteria. For further details on the swimming strategies of unipolar and bipolar flagellated bacteria, we refer the reader to two excellent recent reviews [32,33]. Briefly, in the run-reverse mode, the bacterium can either swim as a pusher, with the flagellar bundle extended behind the body rotating in a counterclockwise manner, or as a puller, when the bundle rotates clockwise and is extended in front of the body. In addition to the extended configuration, the flagellar bundle has also been observed to wrap around the body, providing extra propulsion as a puller [32,33]. After a perfect reversal, the bacterium changes its direction of swimming by ~180°. In the run-reverse-flick, the monotrichous bacterium performs a forward run as a pusher, reverses direction by swimming as a puller, and when it switches back to swim forward, the flagellum flicks by ~90° to select a new direction. The run-reverse-flick mechanism likely involves a buckling instability of the flagellar hook [34]. 

These swimming strategies may be particularly useful in negotiating highly viscous or turbulent environments. The viscoelastic properties of the medium may also play a role in determining the speed of reversal. In a recent study, *E. coli* and *B. subtilis* bacteria swimming in cervical mucus [35] were observed to reverse along the same track they created in the mucus while going forward, swimming faster when returning. This may be related to the fact that mucus is a shear-thinning fluid [36]. As bacteria swim forward, they exert a shear force which reduces the viscosity of mucus. This causes the bacterium to swim at a faster speed when traveling back on the same path created in the forward motion. 

**Modeling bacteria swimming.** The mathematical modeling of the hydrodynamics of swimming bacteria has been the subject of many studies and has been extensively reviewed [37,38]. Here, we briefly capture the essential features for modeling flagella-driven bacterial swimmers. As is well known, the inertial forces on the bacterium are negligible compared to the viscous drag forces exerted by the fluid on the bacterium, i.e., the Reynold’s number, which is the ratio of the inertial force to the viscous drag force, is very small (for the typical helicobacter swimming in an aqueous medium it is about 10^−6^). Under this condition, the swimming is dominated by the viscous forces acting on the bacterium, and the flow of the fluid as the bacterium moves through it can be modeled by the time-independent, linear Stokes equation. Furthermore, the linearity of the Stokes equation implies that the force F and torque T acting on a rigid body are linearly related to the speed V and rotation Ω via a resistance matrix, whose coefficients describe the resistance of the swimmer to the hydrodynamic drag of the fluid and depend on the shape of the body and its orientation. This fact is often used to model the hydrodynamic forces and torques on the cell body by approximating it as an ellipsoid, for which the resistance matrix is known. More complex geometries, such as the helical bodies of *Helicobacter* spp. or bacterial flagella, usually prevent the flow field and resistance matrix from being solved analytically. 

Several theoretical and computational tools have been developed [37] to address this problem. Prominent among these is the Resistive Force Theory (RFT), which decouples the motion of the flagella and cell body and treats the flagellum as a separate thin filament [39]. In the RFT, long-range hydrodynamic interactions are ignored, and the force and torque of each small segment of a filament are proportional to the local speed and angular rotation rate of that object and the anisotropic drag for motion parallel and perpendicular to the filament. The total force and torque on the flagellum can be obtained by adding up the force and torque on each segment. Then, by applying zero total force and torque constraints for a free swimmer in the fluid, assuming a constant angular velocity of the flagellum relative to the cell body, it is possible to calculate the swimming speed and the trajectory of the swimming bacterium. Using the assumption that the flagellar thickness is small compared to its length, the Resistive Force Theory can be improved by including long-ranged hydrodynamic interactions in a method called the Slender Body Theory [37]. However, that approximation makes it inapplicable for many scenarios.

A common group of methods that include all hydrodynamic interactions and can be applied to any geometry are boundary-element or boundary-integral methods, in which the fluid flows are represented as the superposition of flows caused by layers of forces and force dipoles at the surface of a swimmer. The Method of Regularized Stokeslets (MRS) [40,41] is a frequently used type of boundary method due to its relative ease of implementation [42]. In the context of bacterial swimming, such calculations are performed by discretizing the actual shape of the surface of the cell body and flagellar bundle using the measured shape parameters of the bacterium and its flagellar bundle and numerically computing the sum of fluid flows due to the forces or force dipoles on each discretization element [43,44]. The flagella and cell body together satisfy the force–torque balance, and this enables the calculation of swimming speeds, reorientations, and trajectories, as well as the strengths of the forces and force dipoles at the surface of the swimmer, hence the total fluid flow field that they induce. 

Random-walk models have been proposed for the analysis of the trajectories of swimming bacteria corresponding to run-reverse and run-reverse-flick swimming mechanisms [30] and have also been applied for the analysis of their drift velocity in chemotactic migration [45]. These models describe how altering the time between reorientation events, changes in swimming direction, and swimming speed affect a bacterium’s ability to explore its environment. Theoretical predictions of these models can be compared to the measurements of the distribution of run speeds and reorientation (or turn) angles and reversal frequencies. 

## 2. Motility of *Helicobacter* spp.

In the following, we review the currently available information regarding the motility of three different gastric *Helicobacter* spp. The most well-studied of these three is *H. pylori*, which, as stated earlier, is unipolar and lophotrichous. The other two that have been studied in some detail are the bipolar, lophotrichous *H. suis*, and the bipolar, monotrichous *H. cetorum.*

### 2.1. Dependence of H. pylori Swimming on Cell Shape and Number of Flagella 

The motility and chemotaxis of *H. pylori* have been discussed in earlier reviews focused on explaining the flagella and chemotaxis molecular machinery [19]. Here, we focus on studies in which the swimming behavior of individual cells was tracked using time-resolved microscopic techniques. Early work along these lines includes the examination of the motility of *H. pylori* in viscous polymer solutions to mimic the viscous mucus environment [46] and a comparison with straight–rod *E. coli* to address the effect of the helical shape [47]. Karim et al. [47] showed that the helical bacteria *H. pylori* and *C. jejuni* swim faster than *E. coli* in aqueous cultures, presumably due to their helical body shape [47]. However, comparing across species is complicated as they differ not only in shape but also in the distribution of flagella. *E. coli* have peritrichous flagella while *H. pylori* have unipolar, lophotrichous flagella. To directly explore the effect of the helical shape of *H. pylori*, Martinez et al. [4] used time-resolved phase contrast microscopy to compare the swimming of wild-type, helical *H. pylori* strains with isogenic, straight-rod mutants (*Δcsd4* and *Δcsd6*) which lacked the helical-shape-determining peptidoglycan peptidases, Csd4 or Csd6 [48]. They noted that the helical shape of the cell body confers about a 7–20% advantage in swimming speed, depending on the strain. They also found that the swimming speed of *H. pylori* varies considerably among different strains due to differences in cell size and the number of flagella. The distribution of speeds in any given sample is very broad due to variations in cell size, shape, and the number of flagella, as well as temporal changes in speed during swimming. To examine the effect of varying the number of flagella, they compared the wild-type B128 strain of *H. pylori* (median number of flagella = 3) with its isogenic flagellar mutants, fliO_ΔC_ (median 1 flagellum) and sRNA_T (median 4 flagella). They observed that the speed was increased in the sRNA_T mutant, which has an extra flagellum compared to the wild-type, and decreased in the case of fliO_ΔC_, which has on average only one flagellum [4]. 

Tracking studies show that *H. pylori* exhibit a run-reverse-reorient swimming mechanism [4,17,18,49,50] in aqueous broth as well as in viscous solutions. In the case of *H. pylori*, an unbundled state of tumbling or re-orientation occurs [4,49,50] between the forward and reverse runs. The analysis of trajectories using the methods developed by Theves et al. [30] to obtain the distributions of run speeds, change in orientation angle, and reversal frequency shows that the reversal frequency decreases for *H. pylori* swimming in viscous methylcellulose and porcine gastric mucin (PGM) solutions compared to Brucella broth (BB10) [4]. 

**Figure 2:**
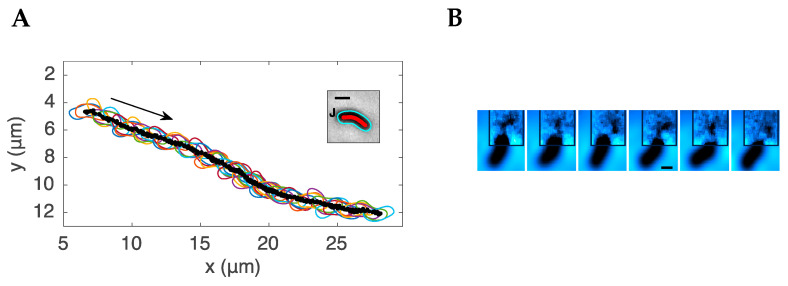
Imaging the detailed motion of a single *H. pylori* bacterium. (**A**). The rotation of the cell body of *H. pylori* is shown by the change in the orientation of successive images of a swimming bacterium. For visual clarity, only the contours are outlined. The inset shows a single bacterium with its contour (blue), central axis (red), and flagellar junction J, outlined using image-analysis tools. (**B**). Consecutive frames of images recorded at 200 fps showing the rotation of the flagellar bundle with a rotation rate of ~66 Hz. The scale bar on both the inset to A and the 4th frame of B is 1 μm. Figure adapted from [49] © The Authors, some rights reserved; exclusive licensee AAAS. Distributed under a Creative Commons Attribution Non Commercial License 4.0 (CC BY-NC).

By imaging *H. pylori* at high magnification (100×) and at 100–200 frames per second, Constantino et al. [49] were able to track the motion of individual bacteria as they swam. The trajectory of a single bacterium at high magnification showed a characteristic oscillation superimposed on the track of its motion (see Figure 2). This feature arises from the rotation of the cell body. To maintain a net zero torque at the flagellar pole, the cell body rotates in the opposite sense relative to the flagellar bundle. The rotation of the cell body can be seen as a change in the relative orientation of its microscopic image as it swims. The rotation of the flagellar bundle of *H. pylori* is also shown in Figure 2. The movies in [49] show the flagellar bundle rotating counterclockwise during the forward run (the bacterium acting as a pusher) and rotating in the opposite sense (clockwise) during the reverse motion, with the bacterium acting as a puller. This study examined only a few bacteria as flagellar bundles show up only occasionally in phase contrast imaging. 

Using an alternative approach by imaging the circular motion of bacteria swimming close to surfaces, Antani et al. [17] showed that *H. pylori* swim at a faster speed when they move as a pusher with flagella rotating counterclockwise, as compared to the reverse run as a puller with flagella rotating in the clockwise rotation. By examining mutants lacking the phosphorylated response regulator CheY-P, they concluded that CheY-P binding to the flagellar motor promotes the clockwise rotation in *H. pylori*. Howitt et al. [18] showed that mutants of *H. pylori* lacking ChePep, a protein that is required for polar localization of some of the chemotaxis components, show more frequent reversals and sustained swimming in the reverse direction, implying that ChePep plays an important role in controlling the directional persistence of motility. 

**Dependence of *H. pylori* swimming speed on the rheology and pH of the medium.** As mentioned in the introduction, *H. pylori* have to swim through mucus in order to colonize the gastric epithelium. In view of this, it is important to examine the effect of the viscosity and pH of the medium on the swimming speed. Studies of *H. pylori* swimming in solutions of the synthetic polymer methylcellulose show that the swimming speed *increases* with increasing viscosity in the low viscosity regime, followed by a decrease at higher viscosities [4,51]. Celli et al. [52] examined the swimming of *H. pylori* in PGM which contains Muc5AC, the major high molecular weight glycoprotein component responsible for the rheological properties of gastric mucus [8,36]. PGM is known to form a gel at pH < 4, while it remains a viscous solution above that pH [36]. Celli et al. [52] observed that, in the absence of urea, *H. pylori* bacteria did not swim in PGM mucin gels at pH 4 and lower, although their flagellar bundle rotated. Upon adding urea, the bacteria were observed to swim, and the pH of the medium went up as indicated by a fluorescent dye in the external medium. Urea hydrolysis produces NH_3,_ which increases the pH of the mucin solution, causing it to de-gel and enabling the bacteria to swim [52]. Thus, *H. pylori* utilize the urease-mediated hydrolysis of urea not only to survive in the highly acidic gastric environment [53] but also to swim in the mucus layer. 

From the study of Celli et al. [52], it is not clear how much of the lack of swimming at low pH is due to the gelation of the medium and how much is due to the effect of varying external pH on the proton-pumping function of the flagellar motors. Su et al. [50] compared the pH-dependent swimming characteristics of *H. pylori* in PGM solutions and BB10 culture broth. They found that in both PGM and BB10, the swimming speed decreased as the pH fell below 4, but in BB10 the bacteria continue to swim at pH < 4, whereas in PGM the bacteria are stuck in various ways to the gel network, their flagella rotate, but they do not swim. They also noted that decreasing pH leads to a decreased fraction of motile bacteria, with a decreased fraction of fast swimmers in the distribution of speeds and net displacement of trajectories. The combination of the effect of pH on the flagellar motors and the sol-gel transition of mucin leads to optimal swimming at a slightly acidic pH of around 5 in porcine gastric mucin. 

**Theoretical modeling of the swimming of *H. pylori*.** Theoretical modeling studies have been used to assess the effect of varying cell shapes and the number of flagella on the swimming behavior of *H. pylori*. A modification of the RFT model developed for elliptical cells [54] was implemented to calculate resistance coefficients for a helical cell body [4]. These calculations showed about a 10% advantage in swimming speed due to the helical shape of the cell body. Varying the motor torque, using a simple linear dependence of motor torque on the number of flagella, produced a larger change in swimming speed, 20–30%, than that produced by only altering the cell shape. The modeling was further refined by Constantino et al. [49] using the more accurate MRS method to calculate the swimming behavior of *H. pylori*. These calculations confirmed that the shape advantage of the helical body in swimming is at most 15%. 

The viscosity of PGM increases by 10-fold or more as the pH drops from 6 to 4 [36]. This implies that the torque exerted by the bacteria flagellar motors would have to increase by an order of magnitude or more to swim in the highly viscous mucin at low pH. Su et al. [50] applied the RFT approach [4] using the measured shape, speed, and body rotation rate to estimate the torque at different pH values in PGM and BB10 in the absence of urea. They showed that the calculated torque is about 10–20 times higher in PGM than in BB10 at pH 6, and about 20–40 times higher in PGM compared to BB10 at pH 4. As the motor torque is directly proportional to the viscosity of the medium, the estimates depend on how much the viscosity of PGM was reduced due to the shear thinning effect from actively swimming bacteria [36]. In order to address how large a variation in torque is feasible for the *H. pylori* flagellar motor, we estimated the maximum torque using the same approach as Beeby et al. [10]. Assuming that the torques generated by all 18 stators are additive, with each stator complex exerting a force of 7.3 pN, and setting the lever arm corresponding to the distance between the outer lobe of the C-ring and the axis of rotation as 27 nm, we estimated a maximum torque of 18 × 7.3 pN × 27nm~3550 pN. nm per flagellum. The unipolar bundle of the *H. pylori* J99 strain used in the study of Su et al. [50] has 1–6 flagella, implying that *H. pylori* J99 can exert 1–6 times the maximal torque per flagellum and that it has the capacity to vary its motor torque by two orders of magnitude by varying the number of active stators (from 1 to 6 × 18 = 108 stators). The faster swimmers are likely to have a larger number of flagella and, consequently, more stator units can be utilized. Furthermore, decreasing the external pH could affect stator function by changing the structure of the protein scaffold of the flagellum in the periplasmic space. The direct influence of pH on these structures would be worth investigating, as would the development of sensors that measure changes in the environmental and intracellular pH while the bacteria are swimming.

### 2.2. Motility of the Bipolarly Flagellated H. suis 

**Figure 3:**
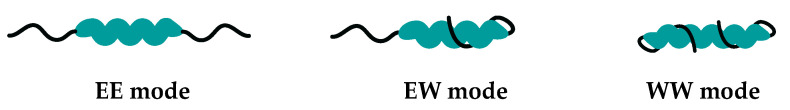
*H. suis* bacterium corresponding to either an extended (E) or a wrapped (W) configuration of the flagellar bundle. The EE mode corresponds to both bundles extended, EW to one extended and the other wrapped, and WW to both wrapped. Adapted from Constantino [57] under a Creative Commons Attribution 4.0 International License.

Studies of several bacteria with bipolar flagella have shown that they swim with one or both flagella extended (E) away from the body acting as a pusher or a puller, or wrapped (W), in which case the flagellar bundle reverses its orientation and rotates while wrapped around the body [32,33,55]. Wrapped flagella have also been observed in unipolar bacteria [56]. For the long and tightly coiled (6–8 turns) bipolar bacterium *H. suis*, Constantino et al. [57] were able to visualize the thick bundles of 8–12 flagella and track their motion by using time-resolved phase contrast microscopy at 100× magnification and frame rates of 100–200 fps. Their study showed that, regardless of media, the flagellar bundles of *H. suis* also assume one of two configurations, E or W. These two configurations correspond to different modes of swimming determined by whether the flagellar bundles at the opposite poles are extended or wrapped around the cell body. This is illustrated schematically in Figure 3. 

Wrapping the flagellar bundle around the body provides additional propulsion. *H. suis* predominantly swim with the lagging flagella extended behind the body and the leading flagella wrapped around the body (EW mode). During a smaller fraction of the runs, *H. suis* swim with both bundles extended away from the body (EE mode) or wrapped around the body (WW mode). However, in the EE or WW modes, the speed was greatly reduced, and the trajectories showed many more reorientations, suggesting that in the EE case, the two flagellar bundles were both acting as pushers and thus negating each other’s action. The fast-swimming EE mode, with one flagellum acting as a pusher and the other as a puller, was not observed. The WW mode could correspond to both flagella acting as pullers in opposite directions. The trajectories in the EW mode are almost linear, whereas, in the WW and EE modes, the bacteria travel lesser distances and display trajectories that show characteristics in between a ballistic and a random-walk motion. Calculations using the Method of Regularized Stokeslets for different modes of the flagellar bundles, including a rolling motion for the wrapped flagellar bundle and the usual pusher-puller mechanisms for the extended bundle, were in qualitatively good agreement with the experimental findings [57]. 

Constantino et al. [57] also observed the flagellar bundle while it changed its orientation axis relative to the cell body, suggesting that there may be a buckling instability in the flagellar hook, or that the hook itself has a flexible joint. However, we note that we have not been able to see flagellar wrapping in the unipolar lophotrichous *H. pylori*. Better methods for visualizing flagella are needed to confirm or refute this finding. A recent study of *C. jejuni*, with fluorescently labeled flagellin, reveals wrapping of the flagella at one or both poles and shows that wrapping enhances the swimming speed in viscous solutions of methylcellulose [58]. This study also shows that the helical shape of the cell body of *C. jejuni* facilitates the unwrapping of flagellar filaments from the cell body upon reversal. 

### 2.3. Motility of H. cetorum, a Monotrichous Bipolar Fusiform Bacterium 

**Figure 4:**
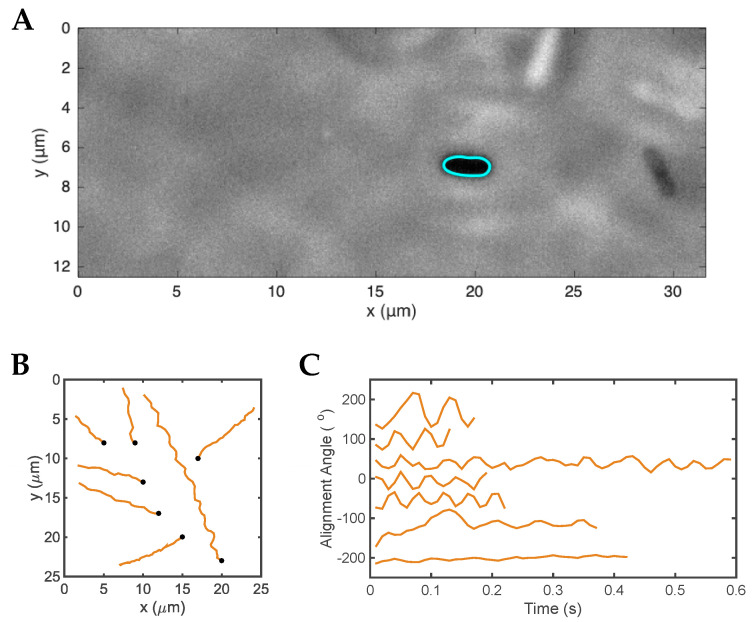
Swimming of *H. cetorum* in culture broth BB10. (**A**) A single frame from the movie shows several bacteria and the contour outlining one of them. (**B**) Trajectories of individual runs. The trajectories were randomly distributed over the figure for better visualization and do not depict the real position in the movie. (**C**) Time-dependent variation of the alignment angle of the center axis of the body relative to the *x*-axis (arbitrarily defined in the movie). The alignment angle values were shifted along the *y*-axis for better visualization. Adapted from Constantino [59].

*H. cetorum* is a fusiform, slightly helical bacterium with bipolar, monotrichous flagella [6]. Constantino [59] reported a live-cell tracking motility study of *H. cetorum* in BB10. The trajectories of *H. cetorum* displayed in Figure 4 resemble those of *H. pylori* and *H. suis*, with the characteristic cell body rotation superimposed on linear tracks. Analysis of the trajectories of *H. cetorum* indicates a tendency to swim in the same direction for long periods without much reorientation over the tracks. The single flagellum of *H. cetorum* is very thin and could not be visualized in time-resolved phase contrast microscopy, unlike the thick bundles of the lophotrichous *H. pylori* and *H. suis* bacteria.

### 2.4. Comparison of the Motility of H. pylori, H. suis, and H. cetorum

**Figure 5:**
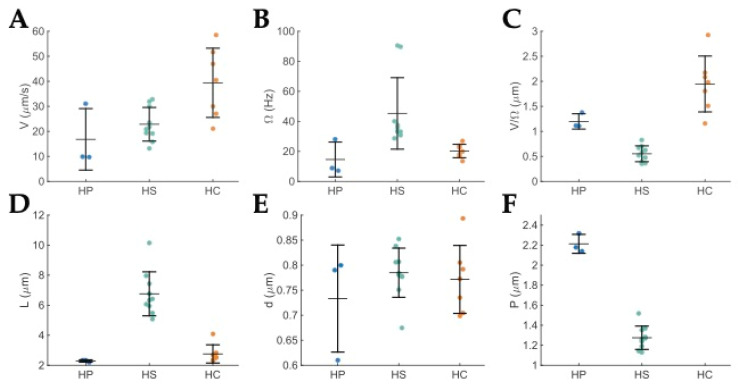
Dot plots of the measured motility and shape parameters for *H. pylori* in blue (HP), *H. suis* in green (HS), and *H. cetorum* in orange (HC) swimming in BB10 broth. The horizontal lines indicate the mean and the vertical lines indicate the standard deviation. (**A**) Average speed, *V* for each run. (**B**) Body rotation rate, *Ω* for each run. (**C**) Distance traveled per revolution, *V/Ω*, for each run. (**D**) Body length, *L* of each bacterium. (**E**) Body diameter, *d* of each bacterium. (**F**) Body pitch, *P* of each bacterium; *H. cetorum* is fusiform, it was only slightly helical, and pitch could not be measured from the images obtained in this study. It can be approximated by the body length. The *H. pylori* data shown here are the same as in [49]. The *H. suis* data correspond to the EW configuration. Adapted from Constantino [59].

A comparison of all the measured swimming and shape parameters for *H. cetorum*, *H. suis*, and *H. pylori* is shown in Figure 5. Table 1 gives the average speed *V*, cell body rotation rate *Ω*, and cell shape parameters: length *L*, diameter *d*, and pitch *P* for these three *Helicobacter* spp. For this comparison, we utilized the *H. suis* data swimming in EW configuration because that corresponds to an optimum run [57].

The fastest body rotation rate was seen in *H. suis.* This is consistent with it having the largest number of flagella and the additional propulsion of the wrapped bundle in the EW mode of the bipolar swimmer. In addition to the number and location of flagella, the shape and size of the body also affect the swimming speed. The ratio *V*/*Ω* is likely to be independent of the number of flagella. It is equal to the average distance traveled during one complete body revolution. Calculations based on the Method of Regularized Stokeslet for *H. pylori* showed that, for a constant flagellar bundle rotation rate, the swimming speed decreases with increased body length and decreased body pitch [49]. Comparing *H. suis* with *H. pylori*, we noted that *H. suis* is almost three times longer, has about a 60% smaller pitch, and has a similar body diameter. These changes can explain the finding that *V/Ω* is smaller for *H. suis* than for *H. pylori* (see Table 1). The faster swimming speed *V* of *H. suis* compared to *H. pylori* suggests that the increased number of flagella and wrapping of the second bundle more than compensates for the slowing down due to the larger length and smaller pitch. Compared to *H. pylori*, *H. cetorum* is a little longer, has a smaller number of flagella, and has a very slightly helical body, i.e., a longer pitch. The longer length would lead to a *decrease* in *V*/*Ω*, while the longer pitch would lead to an *increase* in *V*/*Ω* for *H. cetorum* compared to *H. pylori*. These factors might explain the larger value of *V*/*Ω* for *H. cetorum* compared to *H. pylori*. 

Surprisingly, *H. cetorum* is *faster* than both *H. pylori* and *H. suis*. This may imply that *H. cetorum* coordinates its flagella at the two poles to give a higher speed, swimming in an EE mode with both flagella extended, one acting as a pusher and the other extended flagellum acting as a puller. Even the EW mode with one extended flagellum acting as a pusher, and the other wrapped flagellum acting as a puller would provide additional propulsion. These issues need to be addressed further by developing imaging techniques that can visualize the thin flagella of *H. cetorum*. The fluorescent labeling of flagellins has been done for the closely related *C. jejuni* and may be a promising approach [58].

A comparison of the swimming of *H. suis* in PGM and culture broth BB10 at different pH values [59] shows that it too exhibits pH-dependent swimming similar to *H. pylori*; *H. suis* exhibits fewer reversals in broth than *H. pylori*. In gastric mucin, *H. suis* bacteria swim the fastest around pH 6, while *H. pylori* show peak swimming speed around pH 5. Both species have a reduced speed and rate of body rotation at pH 4 in gastric mucin and are stuck in the gel phase of mucin. The frequency of reversals decreases in mucin for both *H. suis* and *H. pylori*. Both of these *Helicobacter* spp. swim with increased directional persistence in gastric mucin, resulting in straighter swimming trajectories which may be advantageous in moving across the highly viscous mucus lining of the stomach. 

## 3. Summary and Future Directions

In summary, we note that, like many other flagellated bacteria, the motility of *Helicobacter* spp. depends on the number, location, and arrangement of flagella, as well as on the shape of the cell body. It also varies between strains of the same species due to variation in the number of flagella. Motility studies with mutants having fewer or larger number of flagella directly confirm that the swimming speed correlates with the number of flagella. A comparison of the three *Helicobacter* spp. shows that *H. cetorum* is the fastest swimmer while *H. suis* has the fastest body rotation. While the faster speed and body rotation rate of *H. suis* compared to *H. pylori* are consistent with *H. suis* having a larger number of flagella with a bipolar configuration, the fastest swimming speed of *H. cetorum* is hard to explain and needs further examination. The motility of the *Helicobacter* spp. depends on the pH of the medium and shows a dramatic reduction at low pH in gastric mucin, which forms a gel below pH 4. Some preliminary measurements of drift speeds and the fraction of bacteria swimming away from low pH have been reported for *H. pylori* in various pH gradients created in microfluidic geometries [60]. Further work to examine the dependence of swimming speed and trajectories of *Helicobacter* spp. in chemotactic gradients is needed. 

The results described in this review suggest that there is considerable work needed to understand the dependence of the motility of *Helicobacter* spp. on flagellar architecture. Direct imaging of the flagella of these bacteria while they are swimming, using darkfield imaging techniques, or visualizing flagella with flagellins or other flagellar proteins labeled with fluorophores, could provide valuable information on the mechanisms underlying different flagellar conformations during swimming in different modes. The presence of the flagellar sheath in gastric *Helicobacter* spp. is an impediment to the direct labeling of flagella with a fluorescent dye. However, it may be possible to use this strategy with some of the enterohepatic helicobacters, which do not have sheathed flagella, or by using mutant helicobacters lacking a flagellar sheath. Such studies would further advance the field, as would the development of bacteria with mutations in genes encoding a specific flagellar protein, and the labeling of specific proteins in the flagellar machinery. Measurements of flagellar torque and rotation rates would provide much-needed parameters for improved theoretical modeling and the comparison between model calculations and experiments. 

The pH-dependent effects on swimming discussed here were in response to changing external pH. The effect of changing the periplasmic pH by using weak, permeable acids has not been attempted. Studies of motility in native mucus have been difficult due to the poor optical qualities and inhomogeneities in native mucus preparations from stomach tissue. The development of model systems such as gastric organoids and gastric monolayers is a promising approach currently being explored. These could be useful as in vitro mucus-secreting systems for investigating the motility and chemotaxis mechanisms that drive Helicobacters to colonize the gastric mucosa. In addition, advances in time-resolved microscopic endoscopic imaging technologies may eventually lead to dynamic imaging of these pathogens in vivo. 

## Figures and Tables

**Table 1:** Average speed *V*, cell body rotation rate *Ω*, and cell shape parameters: length *L*, diameter *d*, pitch *P* of *H. pylori*, *H. suis*, and *H. cetorum* in BB10 broth. The *H. suis* data are for the EW case, as runs can only be observed in that mode.

	*H. pylori*	*H. suis*	*H. cetorum*
*V* (μm/s)	17 ± 12	23 ± 7	39 ± 14
*Ω* (s^−1^)	15 ± 12	45± 24	20 ± 4
*V*/*Ω* (μm)	1.2 ± 0.2	0.6 ±0.2	1.9 ± 0.6
*L* (μm)	2.29 ± 0.08	7 ± 1	2.8 ± 0.6
*d* (μm)	0.7 ± 0.1	0.8 ± 0.05	0.77 ± 0.07
*P* (μm)	2.21 ± 0.09	0.8 ± 0.07	Not measured

## Data Availability

Not Applicable.
